# Correction: SuFEx-enabled, chemoselective synthesis of triflates, triflamides and triflimidates

**DOI:** 10.1039/d2sc90060b

**Published:** 2022-03-22

**Authors:** Bing-Yu Li, Lauren Voets, Ruben Van Lommel, Fien Hoppenbrouwers, Mercedes Alonso, Steven H. L. Verhelst, Wim M. De Borggraeve, Joachim Demaerel

**Affiliations:** Molecular Design and Synthesis, Department of Chemistry, KU Leuven Celestijnenlaan 200F, Box 2404 3001 Leuven Belgium joachim.demaerel@kuleuven.be wim.deborggraeve@kuleuven.be; Eenheid Algemene Chemie (ALGC), Department of Chemistry, Vrije Universiteit Brussel (VUB) Pleinlaan 2 1050 Brussels Belgium; Laboratory of Chemical Biology, Department of Cellular and Molecular Medicine, KU Leuven O&N I bis, Herestraat 49, box 901 3000 Leuven Belgium; Leibniz Institute for Analytical Sciences ISAS e.V., Otto-Hahn-Str. 6b 44227 Dortmund Germany

## Abstract

Correction for ‘SuFEx-enabled, chemoselective synthesis of triflates, triflamides and triflimidates’ by Bing-Yu Li *et al.*, *Chem. Sci.*, 2022, **13**, 2270–2279, DOI: 10.1039/D1SC06267K.

The authors would like to correct the second sentence in the Acknowledgements section, which should read “For HPLC analyses, we kindly acknowledge Marcus Frings and the support from RWTH Aachen University.”

The Royal Society of Chemistry apologises for these errors and any consequent inconvenience to authors and readers.

## Supplementary Material

